# Downregulation of PUMA underlies resistance to FGFR1 inhibitors in the stem cell leukemia/lymphoma syndrome

**DOI:** 10.1038/s41419-020-03098-1

**Published:** 2020-10-20

**Authors:** Yun Liu, Baohuan Cai, Yating Chong, Hualei Zhang, Chesley-Anne Kemp, Sumin Lu, Chang-Sheng Chang, Mingqiang Ren, John K. Cowell, Tianxiang Hu

**Affiliations:** 1 Georgia Cancer Center, Augusta University, Augusta, GA 30912 USA; 2 Department of Geriatrics, Union Hospital, Tongji Medical College, Huazhong University of Science and Technology, Wuhan, China; 3 Department of Pediatrics, Tongji Hospital, Tongji Medical College, Huazhong University of Science and Technology, Wuhan, China; 4 Department of Radiation Oncology, Tianjin Medical University Cancer Institute and Hospital, National Clinical Research Center for Cancer, Key Laboratory of Cancer Prevention and Therapy, Tianjin’s Clinical Research Center for Cancer, Tianjin, China; 5 College of Allied Health Sciences, Augusta University, Augusta, GA 30912 USA; 6 Consortium for Health and Military Performance (CHAMP), Department of Military and Emergency Medicine, Uniformed Services University of the Health Sciences, Bethesda, MD USA

**Keywords:** Myeloproliferative disease, Apoptosis

## Abstract

Resistance to molecular therapies frequently occur due to genetic changes affecting the targeted pathway. In myeloid and lymphoid leukemias/lymphomas resulting from constitutive activation of FGFR1 kinases, resistance has been shown to be due either to mutations in FGFR1 or deletions of PTEN. RNA-Seq analysis of the resistant clones demonstrates expression changes in cell death pathways centering on the p53 upregulated modulator of apoptosis (Puma) protein. Treatment with different tyrosine kinase inhibitors (TKIs) revealed that, in both FGFR1 mutation and Pten deletion-mediated resistance, sustained Akt activation in resistant cells leads to compromised Puma activation, resulting in suppression of TKI-induced apoptosis. This suppression of Puma is achieved as a result of sequestration of inactivated p-Foxo3a in the cytoplasm. CRISPR/Cas9 mediated knockout of Puma in leukemic cells led to an increased drug resistance in the knockout cells demonstrating a direct role in TKI resistance. Since Puma promotes cell death by targeting Bcl2, TKI-resistant cells showed high Bcl2 levels and targeting Bcl2 with Venetoclax (ABT199) led to increased apoptosis in these cells. In vivo treatment of mice xenografted with resistant cells using ABT199 suppressed leukemogenesis and led to prolonged survival. This in-depth survey of the underlying genetic mechanisms of resistance has identified a potential means of treating FGFR1-driven malignancies that are resistant to FGFR1 inhibitors.

## Introduction

Tyrosine kinase inhibitors have proven a highly effective approach to the treatment of a wide variety of cancers^1^ but, as mono therapies, resistant clones almost inevitably emerge requiring alternative approaches to treat these resistant derivatives^2^. The stem cell leukemia/lymphoma syndrome (SCLL) is an aggressive leukemia subtype that presents with myeloproliferative disease which progresses to AML and which can be accompanied by T-cell and B-cell leukemias/lymphomas^3^. SCLL is characterized by the constitutive activation of FGFR1 kinase resulting from chromosome translocations that create chimeric proteins, where the partner genes provide dimerization motifs to the chimeric kinase domain. As a result, the chimeric kinases are constitutively activated and signal to downstream pathways that promote leukemogenesis.

FGFR1 inhibitors have been developed over the past decade which show greater or lesser specificity for their target and which have proven effective in suppressing SCLL cell growth in vitro as well as leukemogenesis in animal models^4^. Although SCLL is a rare disease, isolated reports demonstrate that FGFR1 inhibitors also have efficacy in human patients with this disease^5^. The lack of data from widespread clinical trials in SCLL, however, have precluded investigations to address the emergence of resistance to these inhibitors but, in murine models, step wise increases in FGFR1 inhibitor concentrations have led to drug resistance in a variety of cell lines expressing chimeric FGFR1 kinases in vitro^6^. These studies, when extended to xenografts studies in vivo, support the idea that resistant cells are insensitive to these inhibitors. The mechanisms of inhibition were shown to be due, so far, to mutations in the FGFR1 domains that affect the ATP-binding sites or deletions within PTEN leading to gene inactivation^7^. Beyond these initial observations, little is known about the mechanisms of resistance, which might provide insights into alternative strategies for treatment of the resistant disease.

In this study, we used RNA-Seq approaches to investigate changes in gene expression associated with the acquisition of resistance to FGFR1 inhibitors in SCLL cells. A consistent finding is the downregulation of the Bcl2 binding component 3 (Bbc3) gene, which is translated into the Puma protein, regardless of the different underlying mechanisms of resistance. TKI treatment, therefore, fails to induce Puma activation which leads to impaired apoptosis in the resistant cells. We have further shown that targeting Bcl2 with the Venetoclax (ABT199) inhibitor provides an option to treat the TKI resistance and suppress leukemic development by the resistant cells in in vivo murine xenograft models.

## Results

### Dysregulation of apoptosis pathways in TKI resistant cells

**Fig. 1:**
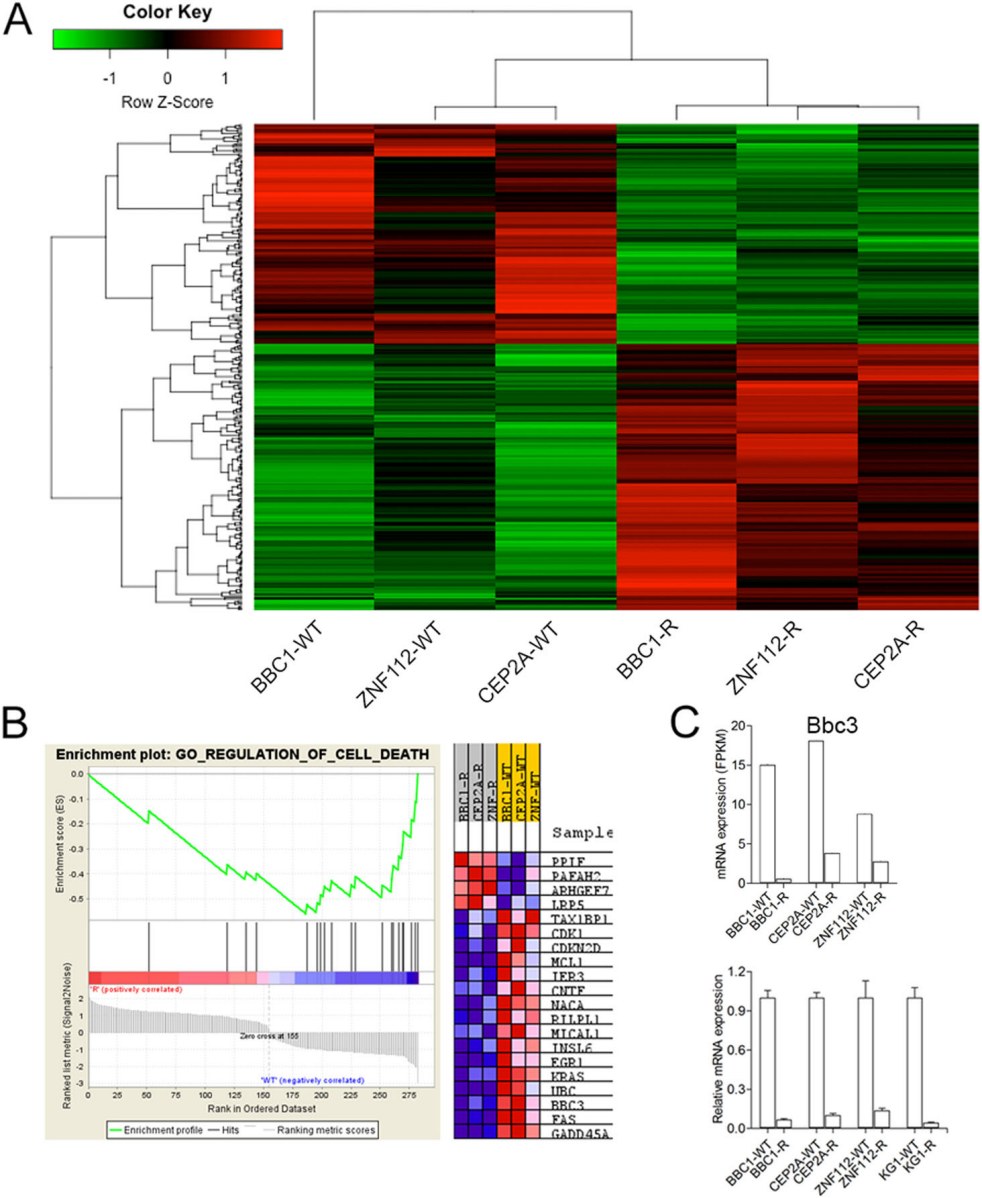
Identification of Bbc3 gene in TKI resistance of SCLL. Hierarchical clustering of RNA-Seq data (**A**), identifies differentially expressed genes between the parental and resistant clones from three mouse SCLL cell lines. GSEA analysis (**B**) identifies genes involved in regulation of cell death within the heat map in (A). The fragments per kilobase million (FPKM) from RNA-Seq for Bbc3 in the different cell lines are shown in (**C**, above), which were consistent with the RT-PCR analyses of the same cells (**C**, below), both showing downregulation of the Bbc3 expression levels in the resistant cells compared with the parental (WT) cells.

Continuous, progressive exposure of SCLL cells to ponatinib resulted in 1000–3000 fold increased resistance levels^7^. Five cell lines carrying different chimeric kinases were studied; mouse lines BBC1 and BBC2 express BCR-FGFR1^8^, ZNF112 expresses ZMYM2-FGFR1^9^, and CEP2A expresses CNTRL-FGFR1^10^. The human KG1 cell line^11^ expresses FGFR1OP2-FGFR1^12^. In all of these cells lines, even though resistance was selected through exposure to ponatinib, pan-resistance for a range of FGFR1 inhibitors was seen, including BGJ398, which we have shown is the most effective inhibitor in this system^6^. BBC2 and KG1 are resistant due to V561M mutations in the ATP-binding domain in the FGFR1 kinase and ZNF112 and CEP2A show inactivating deletions of Pten^7^. To evaluate the gene expression changes that accompany the resistant phenotype, we used RNA-Seq and defined a subset of genes which were consistently either upregulated or downregulated in the resistant cells (Fig. 1A and Supplementary Table 1). Gene set enrichment analysis (GSEA) demonstrated regulation of cell death (Fig. 1B) as the most significantly altered pathway which, in particular, included downregulation of the Bbc3 gene, which translates into the Puma protein. RT-PCR analysis confirmed the down regulation of Bbc3 in the different SCLL cell lines (Fig. 1C) demonstrating a consistent change in all SCLL cell lines examined regardless of the underlying mechanism of resistance. Since Ingenuity Pathways Analysis of relative gene expression data from resistant and parental cells also indicates a crucial role of Puma (Supplementary Fig. 1), we focused on its role in drug resistance.

### PUMA activation and cell apoptosis is impaired in resistant cells

**Fig. 2:**
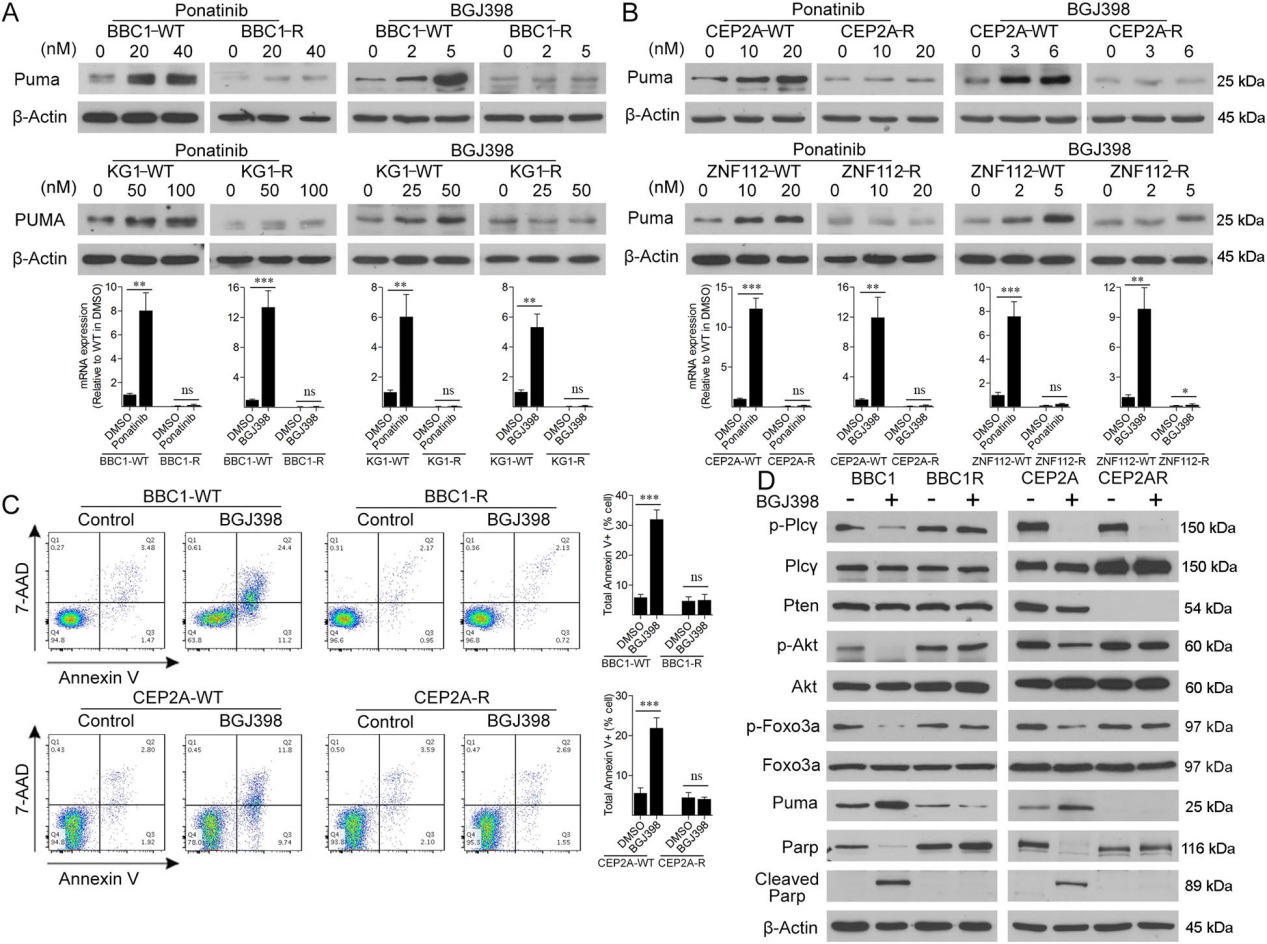
Imparied Puma activation contributes to TKI resitance SCLL. Western blot analysis of PUMA protein levels in the murine SCLL BBC1 cell line (**A**, above) or human KG1 cell line, after treatment at the indicated concentrations of either the ponatinib or BGJ398 FGFR1 inhibitors, show a dose dependent increase in the parental cells, while no changes are seen in the resistant cells that carry FGFR1 mutations in the inhibitor binding site. The same effect is seen in in cell lines CEP2A and ZNF112, which are resistant due to Pten deletions (**B**, above). RT-PCR analysis in all four cell lines shows that transcription levels of PUMA increases significantly in the cells treated (**A**, **B**, below) with the FGFR1 inhibitors at the maximum concentrations shown in A and B (above). Flow cytometric analysis of BBC1 and CEP2A cells shows an increase in 7AAD/Annexin V+ cells in the parental cells treated with BGJ398 at the IC50, but no changes are observed in the resistant cells (**C**). Western blot analysis (**D**) of signaling intermediates demonstrates consistent Akt activation and Puma suppression in the resistant cells (see text) and phosphorylation of Foxo3a is suppressed in parental, but not in the resistant cells treated with BGJ398. Student’s *t*-test was used for statistical comparison between groups. **p < 0.01; ***p < 0.001; ns not significant.

When all four cell lines BBC1, KG1, CEP2A, and ZNF112 were treated with either ponatinib or BGJ398, there is a dose-dependent increase in PUMA expression (Fig. 2A, B, above). When BBC1-R or KG1-R resistant cells, where resistance results from FGFR1 mutation, were treated with either ponatinib or BGJ398, there are no changes in PUMA levels (Fig. 2A), implying suppression of FGFR1 activity induces PUMA activation. RT-PCR analysis in these cells confirms significant increases in PUMA mRNA levels following treatment with FGFR1 inhibitors (Fig. 2A, below). The same response to FGFR1 inhibitors was seen in the ZNF112-R or CEP2A-R resistant cells (Fig. 2B, above), in which resistance was accompanied by Pten deletions. RT-PCR analysis of these cells again shows that the FGFR1 inhibitors leads to increased transcription of Puma (Fig. 2B, below). Flow cytometric analysis of either BBC1 or CEP2A cells, treated with BGJ398, demonstrates increased levels of Annexin V+ cells in parental cells (Fig. 2C), indicating increased levels of apoptosis following TKI treatment. In the resistant cells, however, BGJ398 treatment failed to induce cellular apoptosis.

To investigate downstream signaling, we characterized key intermediates resulting from FGFR1 activation (Fig. 2D) in parental and resistant cells, which developed either through FGFR1 mutations (BBC1) or Pten deletions (CEP2A). In BBC1, levels of Plc-γ, a downstream target of FGFR1, decline in parental cells after BGJ398 treatment as expected but not in the resistant cells since levels of FGFR1 activation remain high. In CEP2A, however, since the cause of resistance is deletion of Pten, Plc-γ expression is suppressed in both parental and resistant cells after BGJ398 treatment, indicating active FGFR1 signaling. In BBC1 parental cells, suppression of FGFR1 activity by BGJ398 leads to reduced p-Akt levels but in the resistant cells, there is no change in p-Akt due to V561M mutations. In CEP2A cells, as previous reported^7^, Pten is not expressed in the resistant cells. Since Akt activation is suppressed by Pten^13^, p-Akt levels are also unaffected in the Pten-deleted resistant cells, compared to a reduction in Akt levels in CEP2A parental cells following drug treatment. The relationship between Puma levels and FGFR1 activity is maintained in BBC1 and CEP2A treated parental cells where reduced activity of FGFR1 leads to increased Puma levels. In the resistant BBC1 cells, since FGFR1 activity remains high in the parental cells, Puma levels remain low in treated cells. In the CEP2A cells, high levels of p-Akt resulting from deletion of Pten, is associated with loss of Puma (Fig. 2D). Thus, in the resistant cells, despite the different mechanisms leading to resistance, the maintenance of high p-Akt levels correlate with low Puma levels. In the absence of Puma, which normally promotes apoptosis, both resistant BBC1 and CEP2A cells do not show cleaved Parp, unlike the parental cells which show increased Parp cleavage following suppression of FGFR1 activation, which is consistent with the increased levels of apoptosis in these cells (Fig. 2C).

**Fig. 3:**
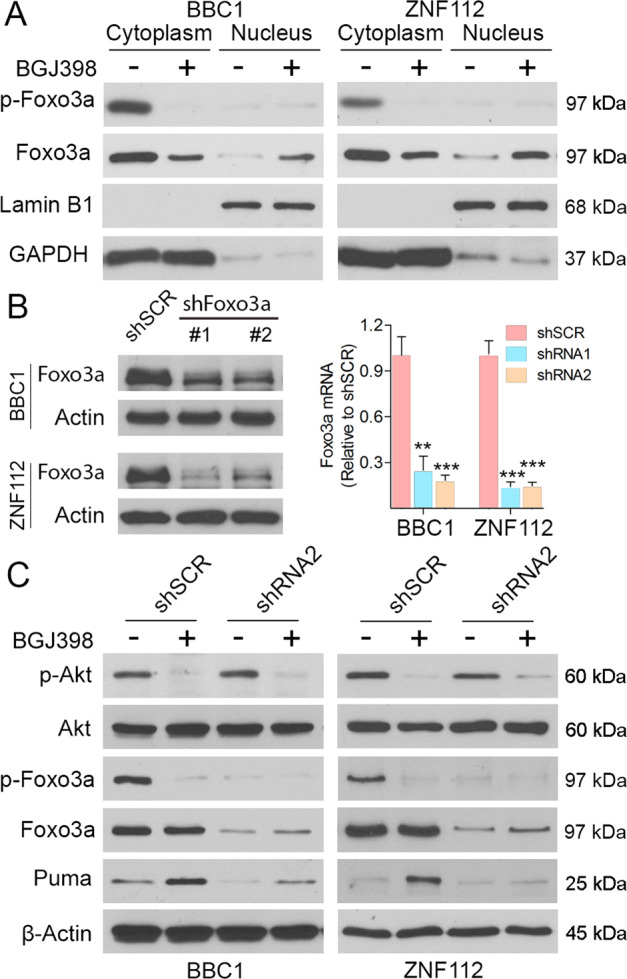
Foxo3a is responsible for Puma activation during TKI treatment in SCLL. Analysis of inactive p-Foxo3a protein levels in BBC1 and ZNF112 parental cells in cytoplasmic and nuclear extracts (**A**) show predominant location in the cytoplasm in untreated cells. Laminin B1 and GAPDH levels indicate the purity of the cytoplasmic and nuclear extracts, respectively. Knockdown of Foxo3a in BBC1 and ZNF112 cells using two individual shRNAs (**B**) shows a more efficient knockdown for shRNA #2 compared with a scrabbled shRNA (shSCR). Compared with shRNA#2 Foxo3a knockdown cells, the shSCR cells treated with BGJ398 displayed reduced Akt activation, reduction of phosphorylated Foxo3a and increased Puma levels, which is impaired in the Foxo3a knockdown cells (**C**). Student’s *t*-test was used for statistical comparison between groups. **p < 0.01; ***p < 0.001; ns not significant.

Puma can be regulated by the Foxo3a transcription factor^14^ and in the BBC1 and CEP2A parental cells, inhibition of FGFR1 function using BGJ398 leads to substantial reduction in Foxo3a phosphorylation levels but there is no effect in the resistant cells (Fig. 2D). We also analyzed the subcellular distribution of the protein when parental cells were treated with BGJ398 (Fig. 3A). In both BBC1 and ZNF112 cells, inactive p-Foxo3a is predominantly in the cytoplasm in untreated cells and suppression of FGFR1 in these cells leads to loss of Foxo3a phosphorylation. Foxo3a is known to be inactivated by Akt phosphorylation^14^ and, as shown in Fig. 2D, inactive p-Foxo3a is only seen when p-Akt is present, suggesting the same relationship between these two proteins is also operating in our system.

To further investigate the role of Foxo3a in Puma activation, we generated Foxo3 knockdown cell lines using two different shRNAs and compared the effects in cells expressing a scrambled shRNA (shSCR). ShRNA #2 showed a more significant knockdown, which is seen at both the protein and transcription levels (Fig. 3B). Analysis of the Foxo3a/Akt axis following suppression of FGFR1 activity, both BBC1 and ZNF112 cells expressing shSRC show the same inhibition of p-Akt and p-Foxo3a, and activation of Puma following pharmacological suppression of FGFR1 function, while in shRNA#2 expressing cells, inhibition of p-Akt by FGFR1 inhibitor failed to induce Puma expression in the absence of Foxo3a, indicating that Foxo3a is indeed operating downstream of Akt in regulation of Puma expression (Fig. 3C).

### Deletion of PUMA protects from TKI-induced apoptosis

**Fig. 4:**
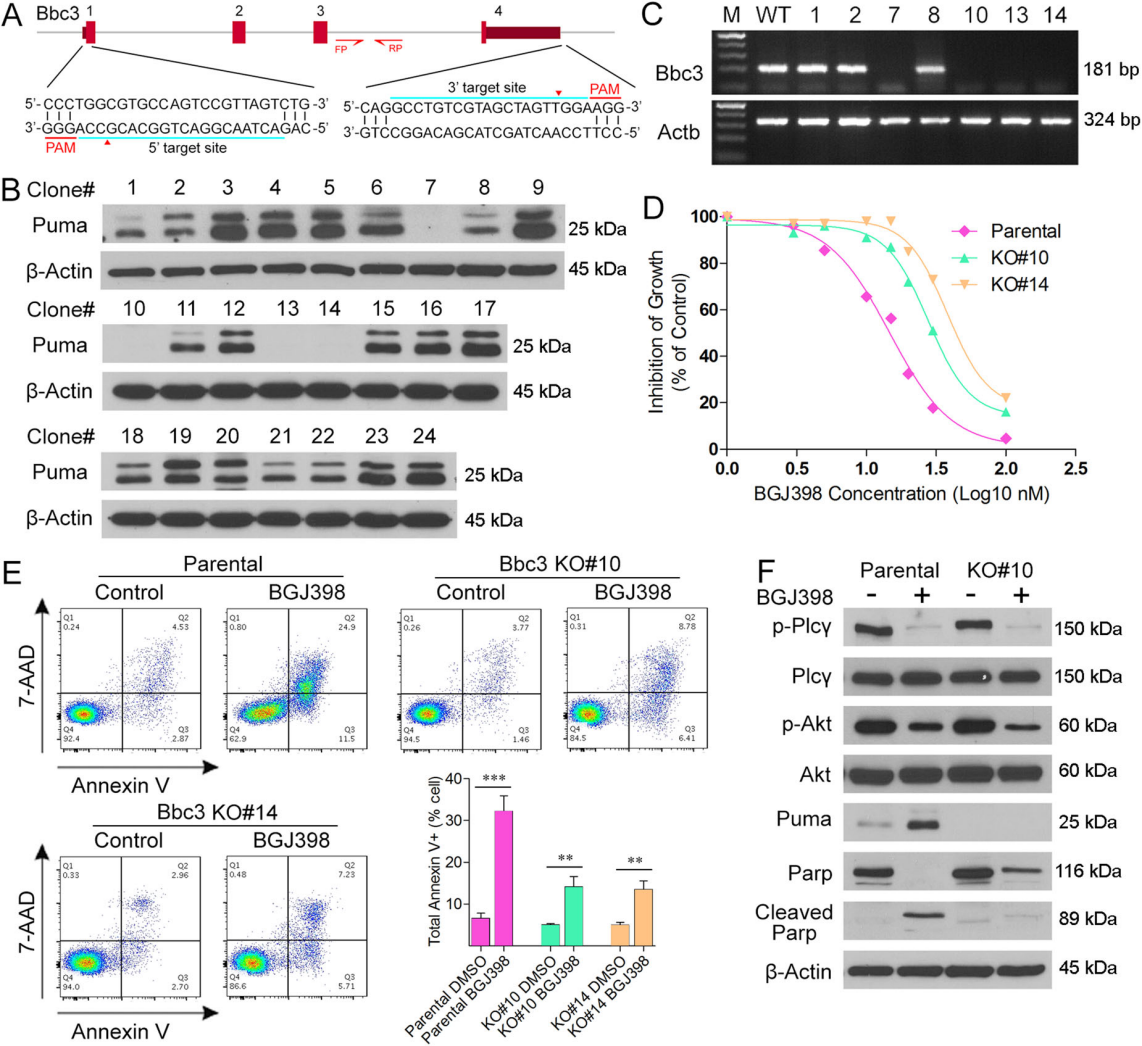
Knockout of Bbc3 gene leads to increased resistance to TKI in SCLL. Schematic diagram of the Puma encoding gene (Bbc3) structure, showing the CRISPR target sites and location of primer pairs to demonstrate deletion of the gene (**A**). Western blot analysis of 24 individual clones identified four (#7, #10, #13, and #14) with complete depletion of Puma protein (**B**) indicating successful knockout. PCR analysis of the Bbc3 locus confirms the deletion in all four cases (**C**). Inhibition of growth by BGJ398, as a percentage of DMSO treated controls (**D**), shows reduced sensitivity in two knockout clones (#10 and #14). IC50 for parental cells = 13.6 nM, for KO#10 = 36.3 nM and for KO#14 = 52.5 nM. Flow cytometry analysis of 7AAD/Annexin V expression levels (**E**) shows reduced induction of apoptosis in the knockout cells. Western blot analysis of downstream signaling in the knockout clones shows loss of Parp cleavage in the KO cells (see text). Student’s *t*-test was used for statistical comparisons between groups. **p < 0.01; ***p < 0.001.

To study the role of Puma in TKI resistance of SCLL cells further, we used CRISPR/Cas9 to delete the gene in BBC2 cells (Fig. 4A). Of 24 single clones analyzed by Western blotting, four showed complete depletion of Puma (Fig. 4B), which were verified as homozygous deletions using locus-specific PCR analysis (Fig. 4C). Cell viability analysis in BBC2 parental cells and knockout clones #10 and #14, treated with increasing concentration of BGJ398, demonstrated that loss of Puma led to reduced sensitivity to FGFR1 inhibition (Fig. 4D). Flow cytometric analysis confirmed that this effect was due to suppression of inhibitor induced cell apoptosis (Fig. 4E), as observed in the cells resistant to FGFR1 inhibitors.

Analysis of downstream signaling, again shows the suppression of Plc-γ activation in the presence of FGFR1 inhibitor in both parental and KO cells, as a result of drug treatment. Activated p-Akt levels are also reduced in treated cells in both parental and KO cells, demonstrating a functional FGFR1 signaling cascade is operating. In the parental cells, Puma levels increase as FGFR1 activation is suppressed, which is accompanied by increased Parp cleavage. However, the absence of Puma coincides with the absence of cleaved Parp. These observations support the idea that FGFR1 stimulates Akt activation, which in turn represses Puma expression leading to suppression of the apoptosis pathway. TKI treatment reverses this suppression of Puma by FGFR1 fusion kinases to induce cell apoptosis. In the absence of Puma, even though p-Akt levels are suppressed by TKI treatment, induction of cell apoptosis is impaired, accounting for the resistance to TKIs.

### 2.4 Targeting Bcl2 in TKI resistant cells restores apoptosis pathways

**Fig. 5:**
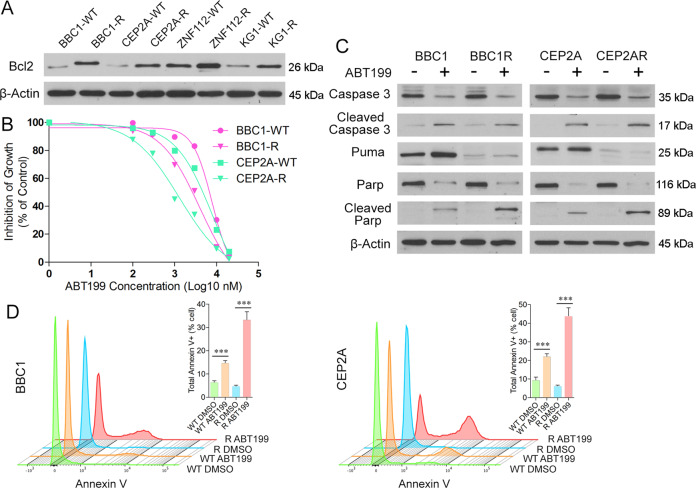
Targeting Bcl2 can onvercome TKI resistance in SCLL. Western blot analysis of relative Bcl2 expression in SCLL cell lines shows increased levels in the resistant derivatives (**A**) compared with parental cells. Treatment of BBC1 and CEP2A with the selective ABT199 Bcl2 inhibitor leads to increased growth suppression in resistant cells (**B**). IC50 for CEP2A-R = 1258.9 nM, for CEP2A-WT–2818.4 nM, for BBC1-R = 5011.9 nM and for BBC1-WT = 7943.3 nM. Increased cleavage of caspase 3 and Parp is seen in the resistant and parental cells (**C**), which is consistent with increased apoptosis in both cases as demonstrated by flow analysis of Annexin V levels (**D**). Student’s *t*-test was used for statistical comparison between groups. ***p < 0.001.

Puma has been shown to downregulate the function of Bcl2 and analysis in the various SCLL cells shows increased Bcl2 levels in the resistant clones from all four cell lines (Fig. 5A). When SCLL cells with either FGFR1 mutations or Pten deletions were treated with the potent ABT199 BCL2 inhibitor^15^, there was a dose-dependent relative decrease in cell survival in both parental cells (Fig. 5B). More importantly, the resistant cells showed an increased sensitivity to ABT199, compared with their respective parental cells. ABT199 treatment of the parental cells was accompanied by the appearance of cleaved caspase 3 and Parp (Fig. 5C), which was also seen in the resistant cells, in the absence of Puma activation. Thus, targeting the pathway downstream of Puma increases cell apoptosis and suppresses cell viability. Consistent with the role of Bcl2 in suppressing apoptosis, flow cytometric analysis shows significantly increased levels of Annexin V in the resistant cells treated with ABT199 in both BBC1 and CEP2A cells, compared to the parental cells (Fig. 5D). These data support the idea that inhibition of Bcl2 could potentially be used as a means of treating SCLL cells that have developed resistance to FGFR1 inhibitors.

### Targeting Bcl2 in vivo leads to suppression of leukemogenesis

**Fig. 6:**
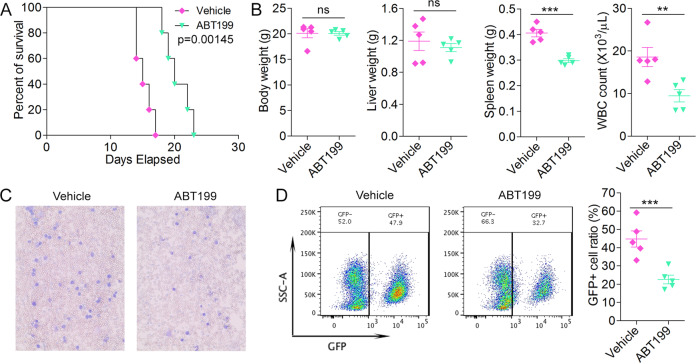
Treatment with Bcl2 inhibitor in vivo improves surivial of leukemic mice with TKI resistance. In vivo analysis of resistant CEP2A-R cells xenografted into BALB/c mice shows a significantly increased survival in cohorts treated with ABT199 (**A**). While no change in the body weight or liver weight was seen at the time of sacrifice, spleen weights and total white blood cell counts were reduced in the treated mice (**B**). Blood smears from drug treated and control mice are shown in C and analysis of the GFP+ leukemic cells in the peripheral blood (**D**) shows a highly significant reduction in the mice treated with ABT199. Images were captured at ×10 magnification using an EVOS FL Auto microscope system. Student’s t-test was used for statistical comparison between groups. *N* = 5 in each group. **p < 0.01; ***p < 0.001; ns not significant.

To explore how ABT199 affects leukemogenesis in vivo, resistant CEP2A-R cells were xenografted into BALB/c mice by injection into the tail vein. Leukemic cell expansion in vivo was allowed for 1 week before starting the drug treatment. Randomly grouped mice were then treated with 100 mg/kg body weight ABT199 via oral gavage daily for 5 consecutive days^16^. As shown in Fig. 6A, mice treated with ABT199 show a significantly enhanced survival, where mean survival increased from 15 to 20 days. This prolonged survival is associated with reduced spleen weight and levels of peripheral blood blast cells, as demonstrated with both white blood cell count and Wright-Giemsa staining of blood smears (Fig. 6B, C). The unchanged body weight indicates low toxicity of this potent BCL2 inhibitor. Since CEP2A-R cells co-express GFP, the impact of ABT199 specifically on leukemic cells is shown in Fig. 6D, where there is a significant reduction in the number of leukemic cells in the peripheral blood in treated mice.

## Discussion

Most TKIs bind the ATP-binding sites in the target protein, preventing phosphoactivation but, it is almost inevitable that resistant clones will emerge that have either mutated the target site preventing binding of the drug or disrupted components of the downstream activation events^1^. In these cases, second line therapies need to be developed as alternative strategies, which makes understanding the mechanism behind the resistance essential. In our studies of drug resistance in FGFR1 driven leukemia/lymphoma syndrome, while mutation of the ATP-binding site was observed, inactivation of Pten was equally common^7^. Our present studies, however, show a common thread in these two distinct mechanisms leading to impaired activation of PUMA. Overexpression of FGFR1 in SCLL leukemic cells stimulates PI3K activity, which leads to p-Akt upregulation^7^. It has been shown that overexpression of AKT suppressed the induction of PUMA-dependent apoptosis^17^. FGFR1 inhibitors, therefore, normally suppress Akt activation in the presence of robust expression of Pten. In cells resistant to FGFR1 inhibitors due to FGFR1 mutation, the kinase function continues to promote Akt activation even in the presence of drug, thereby suppressing Puma whether or not they are treated with inhibitor. Since deletion of Pten also prevents Akt activation, cells with this mechanism of resistance, are still molecularly responsive to FGFR1 inhibitors, but p-Akt is not inhibited. Consequently, Puma is suppressed, and apoptosis prevented. In contrast, the Puma KO cells respond to FGFR1 inhibitors by reducing p-Akt levels, but in the absence of Puma apoptosis is still suppressed. Thus, loss of Puma in the resistance cells, by whatever mechanism, has the same effect on apoptosis.

PUMA is a potent killer from the BCL2 family of proteins^18^ and exerts its influence both through p53-dependent and independent mechanisms^19^. Puma can be induced following treatment with FGFR1 kinase inhibitors, but resistance to these inhibitors keeps Puma levels low, resulting in low levels of apoptosis. PUMA is regulated mostly at the transcription level and a number of different transcription factors have been implicated, most noticeably p53^18^. However, PUMA can also be activated by Myc, which we have shown to be upregulated in SCLL cells as a result of FGFR1 signaling through STAT3^20^. Myc, however, is unlikely to be the means of PUMA upregulation in this case, since inhibition of FGFR1 kinase leads to downregulation of Myc but leads to increased levels of PUMA. Another recognized mechanism of PUMA activation is mediated through FOXO transcription factors, which are phosphorylated by AKT, preventing their concentration in the nucleus^21,22^. Inhibition of PI3K-AKT signaling has also been shown to promote PUMA activity. We now show that Puma expression is dependent on Foxo3a, which in SCLL cells is sequestered in the cytoplasm through phosphorylation by p-Akt. Therefore with the increased p-Akt levels in the resistant cells, whether as a result of FGFR1 mutation or Pten deletion, this Foxo3a mediated Puma activation is impaired.

BIM is another protein in the BCL2 family that has overlapping function with PUMA in promoting apoptosis by binding with BAK and BAX to destabilize the mitochondrial membrane^23–25^. We demonstrated in our original study of FGFR1 inhibitor resistance that Bim was also inactivated in the resistant cells^7^. While BIM can be regulated by posttranslational modification, it can also be regulated at the transcription level and FOXO3A is the key transcriptional regulator^26^. Thus, p-Akt suppression of FOXO transcriptional function may be highly relevant to resistance to FGFR1 inhibitors, since it affects two key regulators of apoptosis. Because of the redundant functions of Puma and Bim in inducing apoptosis, in the absence of Puma, Bim signaling can be activated, possibly explaining why Puma knockout can only partially rescue the induced apoptosis when the knockout clone cells are treated with BGJ398.

Targeted therapies against FGFR1 have proved effective using a variety of different drugs^6,27^ in mouse models of SCLL. In a limited set of patients, FGFR1 inhibitors have also been suggested to be effective^5,28^, although inevitably requiring bone marrow transplants. As FGFR1 inhibitors become more mainstream for SCLL, however, it is likely that resistance will arise and, in these cases, Bcl2 inhibition may provide an alternative therapy. Indeed, venetoclax (ABT199), which was the first selective BCL2 inhibitor to enter routine clinical practice, has already been shown to induces rapid onset of apoptosis in CLL^29,30^. Selective targeting of BCL2 with venetoclax had a manageable safety profile and induces substantial responses in patients with relapsed CLL or Small Lymphocytic Lymphoma, including those with poor prognostic features^31^. In addition, the Brutons tyrosine kinase inhibitor, ibrutinib, increases BCL2 dependence and enhances sensitivity to venetoclax in CLL^32^. We now show that consistent inactivation of PUMA impairs the TKI-induced apoptosis in all SCLL cell models and apoptosis can be induced using the venetoclax BCL2 inhibitor to bypass drug resistance. Thus, targeting Bcl2 in combination with TKIs for SCLL may be a more efficient approach in treating this disease.

## Materials and methods

### Cell analyses in vitro

KG1 cells were purchased from ATCC. Murine cell lines BBC1 BBC2, ZNF112, and CEP2A, were generated in-house^8–10^. The TKI resistant cell lines BBC1-R, ZNF112-R, CEP2A-R and KG-1R were generated as described previously^7^. All cell lines were cultured in RPMI 1640 medium containing 10% FBS, 100 U/mL penicillin, and 100 U/mL streptomycin. Cells were routinely checked by morphology and western blot confirmation of FGFR1 fusion kinase. All of these cell lines were free of mycoplasma contamination.

Drugs used in these studies were: BGJ398, Ponatinib (AP24534), and Venetoclax (ABT199) (Selleckchem, Houston, TX). For drug inhibition assays, cells were treated at the indicated concentrations of BGJ398, Ponatinib or ABT199 for 72 h, followed by cell viability assays using the CellTiter-Glo Luminescent Cell Viability Assay (Promega, Madison, WI). To measure apoptosis, 10^6^ cells were treated with the indicated concentration of drugs for 24 h and then stained with APC Annexin V and DNA binding dye 7-amino-actinomycin (7-AAD) according to the manufacturer’s protocol (Biolegend, San Diego, CA.) and analyzed using BD FACSCanto flow cytometry (BD Bioscience, San Jose, CA). All drug treatment experiments were repeated at least three times, and the representative results were presented.

### Molecular studies

Western blot, genomic DNA preparation, plasmid transfection, shRNA knockdown and quantitative RT-PCR assay procedures are all standard and have been described extensively previously^9,33^. The antibodies used for western blot includes Puma (Cell signaling, #12450), β-Actin (Cell signaling, #5125), p-Plcγ (Invitrogen, #44–696G), total Plcγ (Cell signaling, #2822), Pten (Cell signaling, #9559), p-Akt (Cell signaling, #9271), Akt (Cell signaling, #9272), Parp (Santa Cruz, #sc-8007), Bcl2 (Cell signaling, #15071), Caspase 3 (Cell signaling, #9662), Foxo3a (Cell signaling, # 12829), p-Foxo3a (Cell signaling, # 9466). Preparation of RNA and next generation sequencing analysis was performed as described previously^10^. Knockdown of Foxo3a in BBC1 and ZNF112 cells was achieved by transduction with lentiviral particles prepared from shRNA clone IDs TRCN0000312843 and TRCN0000312844, and then selected with 1 μg/ml puromycin to generate stable cell lines.

### RNA-Seq and data analysis

Preparation of RNA and sequence analysis was performed as described previously^20^. The 283 genes with significant expression change between the resistant and parental cell lines were used to plot the heatmap and details of these genes are provided in Supplementary Table 1.

### CRISPR/Cas9 knockout

For locus-specific deletion of Puma, we designed two guide sgRNAs of 20 nucleotides (nt) targeting the 5′ and 3′ flanking regions of the gene (sgRNA1: 5′- ACTAACGGACTGGCACGCCA -3′; sgRNA2: 5′- GCCTGTCGTAGCTAGTTGGA -3′), using CRISPR Targets Track on Genome Browser^34^. The DNA templates for the sgRNAs were separately inserted into the BbsI and BsaI sites in the pX333 plasmid (Addgene plasmid #64073). The fragment encoding the two guide sgRNAs driven by the U6 promoter was then subcloned into the lentiCRISPR v2 plasmid (Addgene plasmid #5296) through the PacI and NheI sites. Lentivirus was produced by transfecting 293FT cells with lentiCRISPR v2, pLP1, pLP2, and pVSVG vectors using lipofectamine 2000 (Invitrogen, Carlsbad, CA). Virus was harvested and target cells were infected with RetroNectin reagent (Clontech, Mountain View, CA). After 2 days, transduced cells were challenged with 1 μg/ml puromycin to generate stable single clones. Clonal lines were grown from single cells and harvested after ∼3 weeks in culture for Western blot analysis. Genomic DNA analysis using PCR was performed for further validation of complete Bbc3 gene knockout. DNA fragments spanning potential off-target sites (OTS’s) were amplified by PCR from the DNA of the clonal lines and sequenced (McLab), as described previously^35^.

### In vivo studies

Approximately 1 × 10^5^ CEP2A-R resistant cells were injected into the tail veins of 6–8-week-old female BALB/c mice and expansion growth allowed for one week before treatment with either inhibitor or vehicle. The randomly grouped mice were then treated from days 7 to 12 with either vehicle or the ABT199 BCL2 inhibitor at 100 mg/kg body weight via daily oral gavage^16^. Mice were sacrificed in the presence of advanced disease. Blood smears were stained with H&E for detection of blast cells. Flow cytometry was performed for evaluation of engraftment ratio of GFP+ leukemia cell in peripheral blood as described previously^9^. All animal experiments were performed under an approved protocol from the Augusta University Institutional Animal Care and Use Committee.

### Statistical analyses

All statistical analysis was performed using the Student’s *T* test. *p = < 0.01, **p ≤ 0.001, ***p ≤ 0.0001, ****p = 0.00001. ns = not significant. Error bars represent standard deviation.

## Conclusion

Here we demonstrated that downregulation of cell apoptosis pathway protein PUMA is a common phenomenon in different mutation or deletion mediated TKI resistance. The direct role of PUMA in drug resistance was further confirmed by knockout PUMA encoding gene Bbc3 in SCLL cell line. Furthermore, we showed that treatment with BCL2 inhibitor Venetoclax can effectively suppress leukemia progression from TKI resistant cells.

## Supplementary information

Supplemental Table 1

Supplemental Figure 1

## References

[1] Gross S, Rahal R, Stransky N, Lengauer C, Hoeflich KP (2015). Targeting cancer with kinase inhibitors. J. Clin. Invest..

[2] Rosenzweig SA (2018). Acquired resistance to drugs targeting tyrosine kinases. Adv. Cancer Res..

[3] Jackson CC, Medeiros LJ, Miranda RN (2010). 8p11 myeloproliferative syndrome: a review. Hum. Pathol..

[4] Cowell JK, Qin H, Chang CS, Kitamura E, Ren M (2016). A model of BCR-FGFR1 driven human AML in immunocompromised mice. Br. J. Haematol..

[5] Khodadoust MS (2016). Clinical activity of ponatinib in a patient with FGFR1-rearranged mixed-phenotype acute leukemia. Leukemia.

[6] Wu Q (2016). Targeting FGFR1 to suppress leukemogenesis in syndromic and de novo AML in murine models. Oncotarget.

[7] Cowell JK (2017). Mutation in the FGFR1 tyrosine kinase domain or inactivation of PTEN is associated with acquired resistance to FGFR inhibitors in FGFR1-driven leukemia/lymphomas. Int. J. Cancer.

[8] Ren M, Tidwell JA, Sharma S, Cowell JK (2012). Acute progression of BCR-FGFR1 induced murine B-lympho/myeloproliferative disorder suggests involvement of lineages at the pro-B Cell Stage. PLoS ONE.

[9] Ren M, Li X, Cowell JK (2009). Genetic fingerprinting of the development and progression of T-cell lymphoma in a murine model of atypical myeloproliferative disorder initiated by the ZNF198-FGFR1 chimeric tyrosine kinase. Blood.

[10] Ren M, Qin H, Kitamura E, Cowell JK (2013). Disregulation of multiple signaling pathways in the development of myeloid and lymphoid malignancies associated with the CNTRL-FGFR1 fusion kinase in human and mouse models. Blood.

[11] Furley AJ (1986). Divergent molecular phenotypes of KG1 and KG1a myeloid cell lines. Blood.

[12] Qin H, Wu Q, Cowell JK, Ren M (2016). FGFR1OP2-FGFR1 induced myeloid leukemia and T-cell lymphoma in a mouse model. Haematologica.

[13] Georgescu M-M (2010). PTEN tumor suppressor network in PI3K-Akt pathway control. Genes Cancer.

[14] You H (2006). FOXO3a-dependent regulation of Puma in response to cytokine/growth factor withdrawal. J. Exp. Med..

[15] Souers AJ (2013). ABT-199, a potent and selective BCL-2 inhibitor, achieves antitumor activity while sparing platelets. Nat. Med..

[16] Peirs S (2014). ABT-199 mediated inhibition of BCL-2 as a novel therapeutic strategy in T-cell acute lymphoblastic leukemia. Blood.

[17] Qiu W, Leibowitz B, Zhang L, Yu J (2010). Growth factors protect intestinal stem cells from radiation-induced apoptosis by suppressing PUMA through the PI3K/AKT/p53 axis. Oncogene.

[18] Yu J, Zhang L (2008). PUMA, a potent killer with or without p53. Oncogene.

[19] Jeffers JR (2003). PUMA is an essential mediator of p53-dependent and -independent apoptotic pathways. Cancer Cell.

[20] Hu T (2018). FGFR1 fusion kinase regulation of MYC expression drives development of stem cell leukemia/lymphoma syndrome. Leukemia.

[21] Dudgeon C (2010). PUMA induction by FoxO3a mediates the anticancer activities of the broad-range kinase inhibitor UCN-01. Mol. Cancer Ther..

[22] Bean GR (2013). PUMA and BIM are required for oncogene inactivation-induced apoptosis. Sci. Signal..

[23] Garrison SP (2012). Genetically defining the mechanism of PUMA- and Bim-induced apoptosis. Cell Death Differ..

[24] Erlacher M (2006). PUMA cooperates with Bim, the rate-limiting BH3-only protein in cell death during lymphocyte development, in apoptosis induction. J. Exp. Med..

[25] Zhang L-N, Li J-Y, Xu W (2013). A review of the role of PUMA, Noxa and Bim in the tumorigenesis, therapy and drug resistance of chronic lymphocytic leukemia. Cancer Gene Ther..

[26] Dijkers PF, Medema RH, Lammers JW, Koenderman L, Coffer PJ (2000). Expression of the pro-apoptotic Bcl-2 family member Bim is regulated by the forkhead transcription factor FKHR-L1. Curr. Biol..

[27] Ren M, Qin H, Ren R, Cowell JK (2013). Ponatinib suppresses the development of myeloid and lymphoid malignancies associated with FGFR1 abnormalities. Leukemia.

[28] Verstovsek S (2018). Treatment of the myeloid/lymphoid neoplasm with FGFR1 rearrangement with FGFR1 inhibitor. Ann. Oncol..

[29] Anderson MA (2016). The BCL2 selective inhibitor venetoclax induces rapid onset apoptosis of CLL cells in patients via a TP53-independent mechanism. Blood.

[30] Roberts AW, Stilgenbauer S, Seymour JF, Huang DCS (2017). Venetoclax in patients with previously treated chronic lymphocytic leukemia. Clin. Cancer Res..

[31] Roberts AW (2016). Targeting BCL2 with venetoclax in relapsed chronic lymphocytic leukemia. N. Engl. J. Med..

[32] Deng J (2017). Bruton’s tyrosine kinase inhibition increases BCL-2 dependence and enhances sensitivity to venetoclax in chronic lymphocytic leukemia. Leukemia.

[33] Ren M, Cowell JK (2011). Constitutive activation of the Notch pathway in ZNF198-FGFR1-induced T-cell lymphomas provides a novel therapeutic target for this atypical myeloproliferative disease. Blood.

[34] Kent WJ (2002). The human genome browser at UCSC. Genome Res..

[35] Hu T (2017). Long non-coding RNAs transcribed by ERV-9 LTR retrotransposon act in cis to modulate long-range LTR enhancer function. Nucleic Acids Res..

