# Supplementary material for: Downregulation of PUMA underlies resistance to FGFR1 inhibitors in the stem cell leukemia/lymphoma syndrome
Source: Cell Death Dis. 2020 Oct 20;11(10):884. doi: 10.1038/s41419-020-03098-1 (PMC7576156; doi:10.1038/s41419-020-03098-1)
Supplement: Supplementary file 1 — Supplemental Table 1 [file 41419_2020_3098_MOESM1_ESM.pdf]

Supplemental Table 1. The detailed expression data for the 283 genes with significant expression change between the parental and FGFR1 inhibitor resistant cell

gene	BBC1-WT	CEP2A-WT	ZNF-WT	BBC1-R	CEP2A-R	ZNF-R	BBC1 fc	CEP2A fc	ZNF fc	median fc
Fas	8.412	8.276	3.751	0.149	0.950	0.652	-56.2812	-8.71544	-5.7569	-23.584511
Inadl	1.673	1.212	0.490	0.051	0.112	0.339	-32.6103	-10.8584	-1.44483	-14.971162
Bbc3	15.054	18.084	8.823	0.599	3.866	2.753	-25.1141	-4.67742	-3.20489	-10.998793
Egr1	7.699	3.060	3.238	0.323	0.468	1.053	-23.8437	-6.53637	-3.0746	-11.151563
E230016M11Rik	1.599	0.780	0.776	0.129	0.154	0.180	-12.4359	-5.08043	-4.32427	-7.2801898
Runx2	3.172	0.903	0.531	0.256	0.178	0.261	-12.4107	-5.06542	-2.03805	-6.5047046
Rrad	3.600	5.863	2.384	0.312	0.754	1.444	-11.5339	-7.77465	-1.65069	-6.9864035
Ier3	49.356	21.493	18.954	4.601	11.928	7.524	-10.7262	-1.80197	-2.5191	-5.0157397
Slc25a2	1.119	1.099	0.443	0.124	0.346	0.170	-9.00271	-3.17103	-2.60038	-4.9247089
H2-Q7,H2-Q9	74.310	31.624	30.802	8.612	19.618	6.306	-8.62811	-1.61199	-4.88421	-5.0414368
Gadd45a	8.105	9.132	5.956	0.942	1.237	1.981	-8.60146	-7.38182	-3.00746	-6.3302458
Hist1h1c	4.303	6.505	2.315	0.589	0.781	1.973	-7.31201	-8.32651	-1.1734	-5.6039729
Gm19897	0.413	0.507	0.539	0.061	0.187	0.217	-6.8255	-2.70571	-2.48266	-4.0046224
H2-Q2	109.552	42.517	34.353	19.171	19.473	17.611	-5.71438	-2.18343	-1.95063	-3.2828118
Insl6	24.877	13.708	16.013	4.452	7.092	9.475	-5.58803	-1.93275	-1.69009	-3.0702899
Pltp	6.556	6.137	2.783	1.216	2.425	1.223	-5.39218	-2.53108	-2.27597	-3.3997446
Tmem71	4.948	9.823	2.579	0.954	1.496	1.779	-5.1842	-6.5668	-1.44937	-4.4001234
Amy1	0.501	0.441	0.523	0.098	0.255	0.211	-5.11964	-1.73281	-2.48272	-3.1117197
H2-K2	39.010	22.129	17.503	7.698	13.700	6.838	-5.06767	-1.61525	-2.5597	-3.0808759
A130049A11Rik	21.976	13.809	9.850	4.510	8.270	6.422	-4.87288	-1.66982	-1.5338	-2.6921671
Sdcbp2	1.282	2.360	0.563	0.289	0.426	0.354	-4.4337	-5.54717	-1.59278	-3.8578838
Dcdc2b	0.557	0.583	0.328	0.126	0.274	0.206	-4.42062	-2.12882	-1.59163	-2.7136899
Gm10012	3.995	2.104	3.546	0.994	1.607	1.565	-4.01767	-1.3088	-2.26566	-2.530712
Adamts6	3.050	2.908	1.370	0.800	0.824	0.755	-3.81383	-3.53085	-1.81422	-3.052967
Cdo1	2.531	3.988	1.339	0.666	0.599	1.240	-3.79723	-6.65472	-1.07997	-3.8439729
Dram2	17.379	8.739	8.004	4.752	5.997	4.808	-3.65731	-1.45726	-1.6648	-2.2597925
Klf6	8.452	24.914	5.736	2.476	2.941	2.526	-3.41406	-8.47193	-2.27059	-4.7188612
Gm8909	29.092	19.402	16.770	8.568	15.196	9.297	-3.39554	-1.2768	-1.80378	-2.1587061
Mdfic	4.941	6.124	6.741	1.471	1.365	2.799	-3.35863	-4.48704	-2.40842	-3.4180289
Rps26	6313.832	3595.708	3327.746	1894.235	2111.979	2526.317	-3.33318	-1.70253	-1.31723	-2.1176485
Rad52	22.460	17.622	12.324	7.032	10.385	9.148	-3.19386	-1.69689	-1.34709	-2.0792819
Gabre	1.352	1.578	0.769	0.436	0.695	0.403	-3.10235	-2.2715	-1.90741	-2.4270859
4921524J17Rik	18.991	28.874	15.858	6.288	11.600	10.482	-3.01992	-2.48903	-1.51291	-2.3406205
Cept1	25.948	19.219	16.463	8.649	12.870	12.981	-3.00001	-1.49327	-1.26818	-1.9204888
Flot1	21.753	23.403	20.720	7.446	13.708	14.721	-2.92154	-1.70721	-1.40755	-2.012103

Rwdd2a	1.260	1.567	1.529	0.444	0.680	1.038	-2.83818	-2.30468	-1.47298	-2.2052793
Smad7	3.097	4.409	1.362	1.097	0.929	0.555	-2.82174	-4.74682	-2.4553	-3.3412868
Gm6654	54.819	36.698	36.275	19.442	22.267	26.907	-2.81956	-1.64813	-1.34819	-1.9386257
Rab13	2.053	3.147	1.119	0.745	0.422	0.540	-2.75432	-7.45666	-2.07394	-4.094973
Fam13b	29.328	42.848	16.860	10.768	12.807	14.406	-2.72373	-3.3458	-1.17038	-2.4133029
Gch1	17.428	12.019	10.128	6.399	6.976	8.523	-2.72337	-1.72295	-1.18829	-1.8782051
Ifi204	9.712	7.364	5.111	3.581	3.567	1.748	-2.71207	-2.06438	-2.92424	-2.566898
Bambi	2.297	2.856	1.802	0.848	1.685	1.044	-2.70749	-1.69494	-1.72698	-2.043136
Pp2d1	1.184	0.858	0.620	0.444	0.365	0.520	-2.66524	-2.34898	-1.19243	-2.0688829
Eaf2	2.078	2.544	1.118	0.784	0.602	0.950	-2.65101	-4.22783	-1.17702	-2.6852877
4933412E12Rik	14.259	8.532	9.511	5.461	6.664	5.012	-2.61112	-1.28018	-1.89781	-1.9297043
Chmp1b	22.363	24.312	19.214	8.587	13.602	16.372	-2.60433	-1.78734	-1.1736	-1.8550906
Gm7334	39.232	46.257	30.034	15.325	26.685	21.755	-2.55993	-1.73348	-1.38056	-1.8913217
Arhgap18	20.567	18.004	11.661	8.227	8.965	11.238	-2.49987	-2.00822	-1.03767	-1.8485873
Ier5	18.748	30.005	13.298	7.661	4.531	5.316	-2.44713	-6.6216	-2.50166	-3.8567967
Slc25a36	17.367	21.422	9.537	7.120	7.791	8.111	-2.4391	-2.74947	-1.17578	-2.1214485
Lonp2	27.398	19.911	21.265	11.570	12.255	14.583	-2.36796	-1.6247	-1.45822	-1.8169594
Pdp1	8.195	8.928	7.973	3.503	3.352	4.350	-2.33937	-2.66317	-1.83275	-2.2784313
Mcl1	85.805	58.312	47.454	36.790	36.327	35.272	-2.3323	-1.60521	-1.34536	-1.7609547
Gm15787	1.134	1.102	0.542	0.487	0.428	0.371	-2.32941	-2.57807	-1.46258	-2.1233547
Kras	21.331	20.236	17.208	9.180	12.107	12.158	-2.32374	-1.67149	-1.41541	-1.8035481
Fnbp4	70.241	87.858	43.837	30.295	39.502	30.348	-2.3186	-2.22415	-1.44446	-1.995737
Nkiras1	3.333	4.629	2.796	1.452	2.017	2.216	-2.2959	-2.2952	-1.26131	-1.9508052
Gimap9	67.868	45.578	38.666	29.564	26.493	19.963	-2.29565	-1.72035	-1.93692	-1.9843053
Cnot4	21.818	17.186	12.986	9.679	11.570	10.242	-2.25426	-1.48531	-1.26798	-1.6691804
Rpl31	1105.582	1101.452	874.197	490.728	693.806	743.172	-2.25294	-1.58755	-1.17631	-1.6722659
Zfp945	2.948	2.588	3.147	1.324	2.124	1.800	-2.22593	-1.21854	-1.74805	-1.7308404
Gm16675	2.706	2.139	1.755	1.221	1.537	1.070	-2.21637	-1.39174	-1.64032	-1.7494752
AI504432	23.732	29.501	22.739	10.806	10.062	18.665	-2.19609	-2.93189	-1.21828	-2.1154213
Cdkn2d	3.885	9.142	3.696	1.780	2.544	1.872	-2.18287	-3.59429	-1.97498	-2.5840503
Laptm5	128.707	97.732	80.208	60.064	69.741	52.570	-2.14284	-1.40137	-1.52575	-1.6899858
Ubc	15.457	14.439	9.923	7.337	7.053	7.289	-2.10669	-2.04704	-1.36146	-1.8383979
Gm15708	0.694	0.699	0.632	0.330	0.392	0.269	-2.10517	-1.78514	-2.3518	-2.080705
Rilpl1	11.282	7.893	8.743	5.462	4.901	6.280	-2.06557	-1.61054	-1.39219	-1.6894333
Napg	12.653	7.868	8.550	6.163	5.677	6.579	-2.05297	-1.38594	-1.29954	-1.5794839
Zfp51	6.798	6.216	5.798	3.340	4.487	3.894	-2.0356	-1.38529	-1.48901	-1.6366357
Pik3r3	1.959	4.090	3.645	0.964	1.450	1.518	-2.03308	-2.8203	-2.40109	-2.4181548

Rps19	2340.612	2449.870	1933.354	1153.091	1745.518	1280.614	-2.02986	-1.40352	-1.50971	-1.6476959
Cntf	2.611	5.121	2.405	1.300	1.479	0.614	-2.00946	-3.46171	-3.91745	-3.1295406
Slc44a2	61.242	46.031	52.787	30.846	44.423	34.180	-1.9854	-1.03621	-1.54438	-1.5219948
Naca	1002.240	912.544	824.619	513.099	539.149	654.563	-1.95331	-1.69256	-1.2598	-1.635224
Tax1bp1	78.234	61.151	75.454	40.118	56.585	51.057	-1.95009	-1.08069	-1.47782	-1.5028666
4921508A21Rik	1.701	1.878	1.184	0.885	1.107	0.935	-1.92296	-1.69666	-1.26681	-1.6288081
Zfpm1	19.867	17.792	14.173	10.409	12.884	12.131	-1.90873	-1.38086	-1.16838	-1.4859895
Prg4	1.311	1.746	0.873	0.700	0.555	0.700	-1.87382	-3.147	-1.24728	-2.089368
Pnrc2	64.112	75.203	52.419	34.308	41.964	39.695	-1.86874	-1.79207	-1.32055	-1.6604557
Klhl9	20.898	20.167	14.227	11.415	11.629	13.491	-1.83082	-1.73423	-1.05451	-1.5398543
A130077B15Rik	17.486	15.207	12.557	9.606	10.927	10.648	-1.82031	-1.3916	-1.17935	-1.4637539
Gpcpd1	11.577	11.526	8.065	6.442	6.303	5.567	-1.797	-1.82861	-1.44858	-1.6913951
A830080D01Rik	11.701	9.749	7.599	6.717	6.402	6.059	-1.74213	-1.52291	-1.25424	-1.5064265
Nfatc3	33.200	27.829	21.755	19.440	19.487	18.643	-1.70783	-1.42808	-1.16692	-1.4342796
Dgka	26.636	22.163	23.533	15.930	15.996	18.880	-1.6721	-1.38554	-1.24646	-1.4346981
2810403A07Rik	33.350	33.666	29.099	20.100	24.443	23.716	-1.65916	-1.37732	-1.22701	-1.4211628
Mical1	31.546	40.585	26.382	19.638	20.680	14.932	-1.60634	-1.96246	-1.7668	-1.7785339
Wtap	57.690	47.568	49.574	36.229	40.697	37.391	-1.59239	-1.16883	-1.3258	-1.3623427
Ppp1r35	42.325	46.691	44.216	26.705	34.364	30.551	-1.5849	-1.35872	-1.4473	-1.463639
Cdk1	104.934	110.323	98.700	66.710	82.006	62.456	-1.57299	-1.3453	-1.58032	-1.4995328
Asb13	7.127	6.951	8.129	4.535	6.126	4.502	-1.57142	-1.13466	-1.80566	-1.5039117
Cnot7	29.831	34.232	28.578	19.213	25.237	23.584	-1.55264	-1.3564	-1.21175	-1.3735953
Csrnp1	13.102	14.671	10.483	8.450	7.951	5.765	-1.55054	-1.84514	-1.8184	-1.7380278
Rnf14	23.502	24.896	19.349	15.273	17.986	18.226	-1.53882	-1.38419	-1.06163	-1.3282136
Cotl1	22.053	21.689	17.689	14.731	10.153	12.121	-1.49706	-2.13627	-1.45936	-1.6975618
C330024D21Rik	0.521	0.832	0.476	0.350	0.356	0.275	-1.48838	-2.33419	-1.7302	-1.8509218
Gpsm3	45.799	38.035	40.706	30.917	28.230	20.449	-1.48137	-1.34733	-1.99058	-1.6064238
Nrp	0.695	0.858	0.686	0.472	0.489	0.546	-1.47119	-1.75418	-1.257	-1.4941215
Gm11974	49.846	50.276	63.512	34.416	32.486	28.676	-1.44833	-1.54764	-2.21478	-1.7369192
2210408F21Rik	2.053	2.503	3.211	1.437	1.805	1.083	-1.42886	-1.38681	-2.96453	-1.9267358
Ik	101.091	98.622	96.414	73.578	79.125	84.451	-1.37392	-1.24641	-1.14166	-1.253997
Hsdl2	12.098	15.504	14.090	8.845	9.308	8.677	-1.36788	-1.66559	-1.62389	-1.5524517
Cdca3	65.017	72.084	75.831	48.220	60.453	52.984	-1.34833	-1.19239	-1.4312	-1.3239724
Etnk1	20.483	27.524	20.762	15.450	16.947	16.716	-1.32582	-1.62416	-1.24199	-1.3973218
Rhno1	37.168	30.875	40.474	28.122	27.829	26.754	-1.32167	-1.10947	-1.5128	-1.3146479
Gt(ROSA)26Sor	23.054	22.347	24.185	17.467	18.482	13.450	-1.31984	-1.20913	-1.79814	-1.4423723
Il2rg	54.789	52.960	73.591	41.814	46.440	37.243	-1.3103	-1.14041	-1.97599	-1.4755671

Ptp4a2	101.249	110.521	87.225	77.651	69.743	70.131	-1.3039	-1.58468	-1.24374	-1.3774407
Vmn1r58	2.810	3.214	2.746	2.181	2.501	2.481	-1.28844	-1.28528	-1.10694	-1.2268865
Hipk1	35.354	34.856	31.017	27.578	26.025	26.429	-1.28195	-1.33934	-1.1736	-1.2649652
Psmd8	118.493	161.058	141.402	94.150	104.073	104.907	-1.25856	-1.54755	-1.34788	-1.3846623
Zfand5	8.773	8.747	9.087	7.011	6.271	5.567	-1.25138	-1.39488	-1.63226	-1.4261722
Ulk1	13.449	12.085	11.481	10.750	9.818	10.726	-1.2511	-1.23087	-1.07039	-1.1841197
Srsf11	96.020	94.878	87.653	77.441	77.555	70.952	-1.23991	-1.22336	-1.23539	-1.2328854
Stx12	16.101	16.209	15.729	13.150	14.950	13.553	-1.22441	-1.08422	-1.1605	-1.1563752
Srp14	126.693	130.485	134.491	104.086	107.266	96.709	-1.2172	-1.21646	-1.39068	-1.2747805
Zfp207	129.226	142.514	126.651	110.579	116.283	119.303	-1.16863	-1.22558	-1.06159	-1.1519335
Mob4	14.570	16.457	14.690	12.468	9.207	12.443	-1.16862	-1.78751	-1.18061	-1.3789123
Ppil4	29.826	32.247	30.903	26.412	23.114	25.597	-1.12926	-1.39514	-1.20728	-1.2438917
Smndc1	31.826	36.281	30.937	28.365	25.195	25.911	-1.12204	-1.44	-1.19397	-1.2520016
Tfg	42.632	39.857	46.850	38.652	37.162	36.453	-1.10296	-1.07253	-1.28522	-1.1535695
Setdb1	27.911	33.037	27.627	25.411	24.675	24.416	-1.09837	-1.33888	-1.13151	-1.1895828
Ccar1	47.357	53.663	51.903	43.800	44.353	45.172	-1.08121	-1.20989	-1.14902	-1.146706
Susd3	10.276	11.544	13.360	9.546	7.167	5.990	-1.0765	-1.61061	-2.23026	-1.6391224
Pcnp	59.876	60.067	63.097	55.650	53.084	45.838	-1.07594	-1.13154	-1.37654	-1.1946715
Ubl7	40.103	44.906	43.041	38.526	38.548	38.700	-1.04093	-1.16494	-1.11218	-1.106014
Efcab14	11.455	12.026	11.453	12.250	13.869	13.545	1.069358	1.153282	1.182621	1.1350871
Ddrgk1	31.729	27.223	31.833	34.372	36.586	37.236	1.083306	1.343915	1.169723	1.1989811
Dlg1	11.973	11.781	11.703	13.501	13.852	14.381	1.127598	1.175848	1.228832	1.1774261
Lrrc45	15.036	11.305	15.144	17.029	19.551	22.026	1.1326	1.729379	1.454402	1.4387934
Gphn	15.939	11.228	12.808	18.349	16.790	21.562	1.151154	1.495298	1.683477	1.4433098
Cpt2	5.477	3.944	5.310	6.364	7.827	7.745	1.161944	1.984449	1.458554	1.5349821
Zfp963	0.948	0.921	0.797	1.104	1.409	1.818	1.165012	1.530359	2.280985	1.6587853
Tshz1	4.455	3.949	3.421	5.249	5.416	4.867	1.178264	1.371667	1.422427	1.3241191
C230035I16Rik	0.661	0.734	0.728	0.780	1.032	0.962	1.18076	1.4067	1.320633	1.3026978
Ttc21b	5.182	4.553	4.780	6.141	5.816	6.943	1.185006	1.277559	1.452342	1.3049689
Rsph3b	1.120	0.860	1.013	1.337	1.524	1.227	1.19379	1.771767	1.210826	1.3921279
Pofut2	9.309	8.194	9.839	11.141	10.754	12.646	1.196706	1.312417	1.285335	1.2648194
Casd1	6.922	4.467	6.792	8.363	9.393	9.410	1.208173	2.102563	1.385532	1.5654225
Actr5	7.952	7.091	8.486	9.787	8.925	9.423	1.230775	1.258711	1.11043	1.1999718
Sap130	16.609	16.170	15.727	20.569	17.773	21.642	1.238446	1.099128	1.376133	1.2379025
Arhgef7	8.418	7.541	9.780	10.471	11.630	12.245	1.243866	1.542189	1.25197	1.3460086
Blcap	5.667	5.643	7.031	7.093	8.919	8.706	1.251697	1.580609	1.238201	1.3568356
Rspry1	6.346	7.029	7.158	8.129	9.638	9.252	1.280859	1.371272	1.292649	1.3149269

Tex264	14.900	15.037	16.939	19.163	18.134	19.473	1.286125	1.205923	1.14959	1.2138794
Rapgef6	6.939	7.402	6.479	9.034	8.460	9.746	1.301903	1.143012	1.504293	1.3164029
Nap1l1	321.224	328.823	377.505	419.931	509.398	419.980	1.307285	1.549156	1.112515	1.3229854
Prkcsh	53.760	57.897	53.026	71.855	64.088	67.056	1.336602	1.10693	1.264578	1.2360365
Uap1l1	6.048	5.220	6.654	8.196	6.935	9.175	1.355121	1.328528	1.37894	1.3541966
Fancd2	7.317	7.691	8.765	10.111	10.798	10.678	1.381837	1.403979	1.218229	1.3346818
Srebf1	27.589	16.909	25.038	38.482	32.864	39.579	1.394813	1.943639	1.58078	1.6397439
Solh	6.348	5.647	6.496	8.923	8.793	8.051	1.405523	1.556931	1.239411	1.4006218
Pik3r4	4.649	3.774	5.151	6.631	5.903	7.632	1.426292	1.563896	1.481528	1.490572
Ttyh3	20.370	18.792	22.665	29.179	25.725	24.145	1.432463	1.368966	1.065299	1.2889091
Ep400	19.303	20.969	19.845	28.104	23.585	25.210	1.455967	1.12477	1.270372	1.2837032
Lrp5	7.887	9.443	9.027	11.539	12.462	10.127	1.463118	1.31966	1.12177	1.3015161
Pygb	15.967	15.435	12.364	23.381	20.357	26.486	1.464391	1.318878	2.142142	1.6418038
D330050I16Rik	3.256	3.890	4.075	4.791	4.846	5.518	1.471718	1.245824	1.354027	1.3571897
Bud13	9.139	7.927	10.084	13.484	13.145	11.860	1.475468	1.658246	1.176128	1.4366139
Alkbh7	6.656	5.854	8.818	9.906	10.132	13.570	1.488335	1.730636	1.538931	1.585967
Gm16023	0.878	0.764	0.588	1.311	1.076	1.402	1.491953	1.407795	2.384651	1.7614663
Nt5m	5.315	2.654	4.839	7.948	6.815	8.859	1.495474	2.568085	1.830862	1.9648069
Xpo7	7.884	6.431	7.954	11.814	8.864	12.115	1.498363	1.378353	1.523024	1.4665798
Fbxo3	19.626	20.880	15.849	29.528	23.913	25.407	1.504507	1.145239	1.603073	1.4176063
Smarca4	52.488	42.055	58.853	79.239	64.059	70.192	1.509669	1.523221	1.192658	1.408516
Tbc1d5	5.862	3.022	3.763	8.855	6.150	8.671	1.510644	2.035319	2.3045	1.9501545
Rundc1	5.109	3.708	5.830	7.742	6.648	9.079	1.515242	1.792842	1.557405	1.6218294
Gid4	7.873	5.598	6.533	11.946	9.141	11.010	1.5173	1.632959	1.685414	1.6118906
4933421O10Rik	1.062	1.096	1.060	1.616	2.126	1.513	1.522124	1.939476	1.427395	1.629665
Map4k5	1.747	1.220	1.988	2.681	2.829	3.273	1.534274	2.318019	1.646082	1.8327916
BC030867	5.539	5.526	7.152	8.672	7.912	10.473	1.56564	1.431622	1.464244	1.4871688
Tlk1	12.882	14.828	16.352	20.279	19.944	17.285	1.574244	1.345042	1.05704	1.3254422
Ehmt1	12.561	12.732	14.999	20.021	15.998	18.585	1.593928	1.256504	1.239066	1.363166
Ralgapa1	5.580	6.613	7.525	8.903	11.317	17.786	1.595506	1.711145	2.363666	1.8901055
Akap9	7.425	7.139	7.618	11.865	10.321	10.548	1.598087	1.44563	1.384654	1.4761235
Crlf3	19.492	20.219	26.486	31.400	26.659	32.727	1.610928	1.318564	1.23565	1.388381
Kat2a	31.308	25.632	38.440	50.819	44.353	46.255	1.623195	1.730363	1.20328	1.5189463
Fto	12.379	10.620	15.133	20.341	18.317	21.023	1.643174	1.724771	1.389208	1.5857175
Scd2	123.740	100.418	139.494	203.398	177.417	237.410	1.643753	1.766786	1.701937	1.7041586
0610038B21Rik	0.730	0.431	0.505	1.210	0.904	0.993	1.657878	2.097252	1.96701	1.90738
Tlcd1	1.584	1.343	2.484	2.651	4.247	5.960	1.673707	3.162477	2.399264	2.411816

Amigo1	1.293	1.839	1.311	2.180	2.161	2.144	1.685995	1.175336	1.63566	1.4989969
Foxk2	18.874	14.847	17.705	31.932	20.712	26.198	1.691875	1.39507	1.479695	1.522213
Mrs2	5.996	7.165	7.881	10.166	8.697	9.330	1.695584	1.21373	1.183868	1.364394
Pafah2	1.564	1.527	2.506	2.663	3.123	2.945	1.703211	2.045567	1.175139	1.6413057
Bod1l	8.051	6.538	8.279	14.027	9.484	10.748	1.742113	1.450727	1.298197	1.4970124
Ints3	16.668	12.755	19.839	29.113	22.763	24.110	1.746591	1.78474	1.215329	1.5822202
Lipe	2.216	2.260	3.071	3.885	3.554	3.747	1.753452	1.572092	1.219988	1.5151773
Impdh2	113.584	108.408	147.163	202.544	171.944	165.159	1.78321	1.586082	1.122286	1.4971927
Sco1	1.653	0.976	2.318	2.958	3.180	3.550	1.789677	3.259463	1.531895	2.1936784
Cep135	3.198	4.589	4.133	5.753	6.001	6.966	1.799155	1.307686	1.685451	1.5974306
Ttll5	3.939	5.417	5.797	7.131	8.150	9.151	1.810437	1.504429	1.578472	1.6311129
Smc6	12.714	13.656	19.650	23.201	23.192	26.907	1.824827	1.698273	1.369346	1.630815
Ascc3	5.208	4.805	5.681	9.513	6.984	7.463	1.826572	1.453526	1.313601	1.5312331
Zfp46	2.645	2.846	3.348	4.833	3.711	4.289	1.827556	1.303561	1.281237	1.4707844
Ogdh	15.015	14.739	16.886	27.804	18.050	28.257	1.851727	1.224633	1.673347	1.5832356
Trim25	17.548	18.916	18.228	32.560	25.963	22.210	1.855504	1.372591	1.218414	1.48217
Erlin1	6.423	8.943	8.447	12.009	11.740	9.688	1.869704	1.312694	1.146946	1.4431148
Tmed10	28.520	26.531	30.826	53.566	37.259	41.212	1.87816	1.404357	1.336957	1.5398244
Dnajc27	2.905	2.715	3.943	5.507	4.659	6.310	1.89575	1.716075	1.600462	1.7374292
Brip1	3.425	5.067	4.778	6.578	6.670	7.455	1.920513	1.316239	1.560383	1.5990451
Flywch1	5.427	4.446	5.936	10.437	8.410	8.976	1.923306	1.891481	1.512268	1.7756851
Tmem39a	8.158	6.211	11.653	15.794	15.058	12.661	1.935995	2.42453	1.086476	1.815667
Ppif	20.583	13.822	23.878	40.576	30.205	31.468	1.97134	2.185185	1.317862	1.8247957
Crtap	4.364	4.564	6.361	8.694	8.164	9.791	1.992122	1.788733	1.539323	1.7733926
Kif1c	5.835	5.255	5.625	11.684	8.135	9.673	2.002458	1.548031	1.719595	1.7566945
Thap2	2.746	3.565	5.251	5.517	8.500	7.973	2.009148	2.384097	1.518364	1.9705362
Nup188	17.820	19.655	27.088	36.403	30.903	32.669	2.042835	1.57223	1.206014	1.6070264
Atraid	10.808	13.277	12.094	22.167	15.623	15.989	2.051	1.176705	1.322115	1.5166069
Kif24	4.715	3.993	3.755	9.684	6.067	5.623	2.054023	1.519298	1.497458	1.6902596
Adat1	2.160	1.664	3.198	4.458	3.718	4.118	2.063487	2.234696	1.28781	1.8619979
Ankfy1	6.175	8.532	8.970	13.100	11.842	14.746	2.121352	1.387893	1.643978	1.7177409
C920009B18Rik	0.851	0.812	0.928	1.814	1.132	1.446	2.131352	1.394144	1.558362	1.6946193
Dgcr2	14.150	10.178	16.621	30.626	22.811	21.055	2.164343	2.241164	1.266788	1.8907648
Rhot1	8.197	8.971	12.513	17.775	12.808	15.781	2.168498	1.427773	1.261136	1.6191357
Atpaf2	10.354	10.782	14.886	22.480	14.908	20.136	2.171142	1.382604	1.352671	1.6354723
Mettl25	1.989	1.370	3.149	4.321	3.896	4.311	2.172383	2.842957	1.368971	2.1281037
Pomt2	1.783	0.940	2.041	3.907	2.762	3.808	2.190802	2.93965	1.865369	2.3319403

BC068281	2.593	1.727	2.541	5.699	3.762	4.422	2.197761	2.177682	1.74015	2.0385312
Gm16119	0.308	0.437	0.440	0.678	0.660	0.848	2.198897	1.510743	1.928461	1.8793672
AU022252	2.295	2.465	3.660	5.068	4.355	5.153	2.207842	1.766437	1.407797	1.7940254
Hadha	19.038	21.423	28.457	42.173	32.221	34.798	2.215259	1.504035	1.222856	1.6473832
Invs	1.536	0.967	2.262	3.423	2.826	3.879	2.228064	2.923352	1.714999	2.2888049
Nbas	2.294	1.774	3.733	5.264	4.770	7.273	2.295286	2.689046	1.947965	2.3107656
Mrm1	3.213	3.760	6.652	7.397	9.657	12.444	2.302539	2.56827	1.870662	2.2471572
Ercc4	1.386	1.026	1.533	3.198	2.167	3.972	2.307643	2.112518	2.592048	2.3374028
Cog5	5.672	6.479	9.707	13.154	10.370	17.751	2.319172	1.600497	1.828779	1.9161492
Qser1	3.590	5.963	4.820	8.392	8.259	7.204	2.337625	1.385188	1.494677	1.7391633
Zfp72	0.944	0.712	1.070	2.225	1.676	1.685	2.356839	2.352717	1.574546	2.094701
Tmed3	6.320	5.313	9.527	14.940	21.350	14.246	2.363769	4.018732	1.495357	2.6259524
Lig3	7.098	7.608	12.637	17.049	13.182	17.551	2.401783	1.732598	1.388845	1.8410754
Nkiras2	2.765	2.779	3.123	6.652	4.894	6.821	2.405635	1.760971	2.184275	2.1169605
4933404O12Rik	7.885	6.444	9.573	19.063	13.071	10.844	2.417725	2.028273	1.132722	1.8595732
Kif16b	1.830	1.872	2.477	4.449	3.173	4.076	2.431373	1.695259	1.645197	1.9239428
Gnat2	0.513	0.452	0.625	1.264	1.099	0.823	2.4613	2.432148	1.318234	2.0705603
Camk2n2	1.451	1.253	2.223	3.573	2.809	2.674	2.462385	2.242144	1.202892	1.9691406
Gm608	2.449	1.868	4.044	6.150	5.216	4.834	2.510653	2.792042	1.195528	2.1660744
Larp1	28.498	35.208	43.472	71.862	46.331	58.381	2.521676	1.315943	1.342938	1.7268525
Mtmr4	5.702	7.341	9.649	14.624	10.019	13.640	2.564706	1.364799	1.413638	1.7810478
Myh10	16.021	27.454	29.684	42.423	42.304	63.462	2.647883	1.54091	2.137891	2.1088946
Cdk6	8.806	13.620	12.695	23.417	21.287	26.157	2.659214	1.562938	2.060418	2.0941901
Utp14b	2.380	3.480	3.103	6.369	3.776	7.164	2.675748	1.084884	2.308924	2.0231854
Tex2	1.184	2.191	2.616	3.303	3.980	4.542	2.788616	1.816365	1.736078	2.1136864
Coil	4.039	3.487	7.778	11.472	8.574	11.487	2.839993	2.458733	1.476784	2.2585032
Pi4ka	6.721	8.060	10.254	19.592	14.418	15.071	2.915022	1.788775	1.469725	2.0578406
Depdc5	4.699	4.192	7.951	13.905	9.285	11.708	2.959121	2.21487	1.472646	2.2155459
Lpcat1	5.633	3.374	5.009	17.484	8.727	7.768	3.103941	2.586962	1.55088	2.4139275
Gm15760	0.440	0.372	0.953	1.386	1.120	1.395	3.148157	3.011727	1.464044	2.5413095
Zfp526	1.756	2.448	4.373	5.697	6.404	6.157	3.244621	2.616098	1.407966	2.4228951
Rapgef1	8.846	11.509	18.513	28.880	24.557	21.073	3.264619	2.133723	1.138284	2.1788757
Tdg	6.749	10.357	11.412	22.066	20.465	17.086	3.269706	1.975863	1.4972	2.2475899
Lrrc49	1.055	0.992	2.753	3.490	3.987	4.756	3.30752	4.018099	1.727664	3.017761
Fitm2	1.240	0.964	1.258	4.129	2.066	1.863	3.330299	2.143556	1.480107	2.3179873
Plod1	1.306	2.552	2.859	4.361	7.555	5.365	3.339766	2.96047	1.876838	2.7256911
Diap2	1.724	2.020	2.641	5.765	2.977	5.819	3.34339	1.473785	2.20344	2.3402051

Dag1	2.292	3.875	3.661	7.905	5.034	6.835	3.448522	1.298979	1.866884	2.2047947
Akap1	6.863	4.788	12.386	24.283	14.017	17.884	3.538431	2.927592	1.443869	2.636631
E130304I02Rik	0.169	0.144	0.248	0.609	0.413	0.490	3.602151	2.866299	1.980174	2.8162079
Glg1	5.108	10.473	6.326	18.770	10.847	16.046	3.674301	1.035683	2.536361	2.4154479
Slc39a9	3.102	5.157	7.196	11.477	8.200	13.413	3.699771	1.590222	1.864071	2.3846879
Aldh3b1	2.596	4.042	2.583	9.803	6.686	5.932	3.776437	1.653875	2.296693	2.5756682
Iba57	0.530	0.669	1.322	2.053	1.609	2.204	3.87463	2.405619	1.667131	2.6491265
Acaca	7.436	5.059	8.990	29.031	13.890	19.834	3.904033	2.745565	2.206152	2.9519165
Arhgap39	0.915	0.905	2.129	3.600	4.277	6.543	3.934784	4.727339	3.072881	3.911668
Gen1	2.538	3.309	6.423	10.019	6.843	10.321	3.948146	2.067898	1.606917	2.540987
Kcnq1ot1	0.299	0.380	0.868	1.217	1.300	1.005	4.072077	3.420693	1.158591	2.8837871
Nsf	3.092	3.903	6.981	14.641	7.513	15.228	4.734308	1.924664	2.181205	2.9467257
Scd3	0.228	0.284	0.315	1.180	0.667	0.493	5.186505	2.344242	1.564697	3.0318147
4930577N17Rik	0.304	0.686	0.771	1.802	1.012	1.509	5.92513	1.473687	1.958495	3.1191038
Slfn9	3.840	4.661	12.163	24.030	17.043	16.851	6.2583	3.656674	1.385433	3.7668022
Galr3	0.222	0.181	0.521	1.394	0.721	0.769	6.271902	3.989715	1.47537	3.9123289
C2cd2	0.339	0.521	0.774	2.138	1.464	1.368	6.305308	2.810968	1.768109	3.6281285
Fam120c	0.479	0.172	0.794	3.340	1.399	2.163	6.97292	8.143366	2.725439	5.9472413
Hfe	0.444	0.800	1.697	3.204	4.236	3.388	7.217711	5.292834	1.996236	4.8355933
Fads2	12.859	6.137	19.908	97.079	45.723	56.848	7.549257	7.450717	2.855572	5.9518486
Cldn20	0.121	0.052	0.465	1.009	0.831	1.111	8.356036	16.12827	2.389162	8.9578234
Skida1	0.122	0.230	0.457	1.104	0.667	0.627	9.058064	2.902065	1.369647	4.4432588
Mia	0.148	0.064	0.328	1.462	0.410	0.939	9.852725	6.458589	2.862302	6.3912055
Trim65	0.344	0.846	2.472	5.063	4.727	5.246	14.71534	5.588964	2.121976	7.4754277
Sfxn5	0.078	0.073	0.252	1.177	1.661	0.992	15.16402	22.80774	3.936576	13.969447
Gm10390	0.024	0.073	0.222	0.600	0.578	0.353	25.3426	7.884434	1.587852	11.604961
