# Supplementary material for: Downregulation of PUMA underlies resistance to FGFR1 inhibitors in the stem cell leukemia/lymphoma syndrome
Source: Cell Death Dis. 2020 Oct 20;11(10):884. doi: 10.1038/s41419-020-03098-1 (PMC7576156; doi:10.1038/s41419-020-03098-1)
Supplement: Supplementary file 2 — Supplemental Figure 1 [file 41419_2020_3098_MOESM2_ESM.docx]

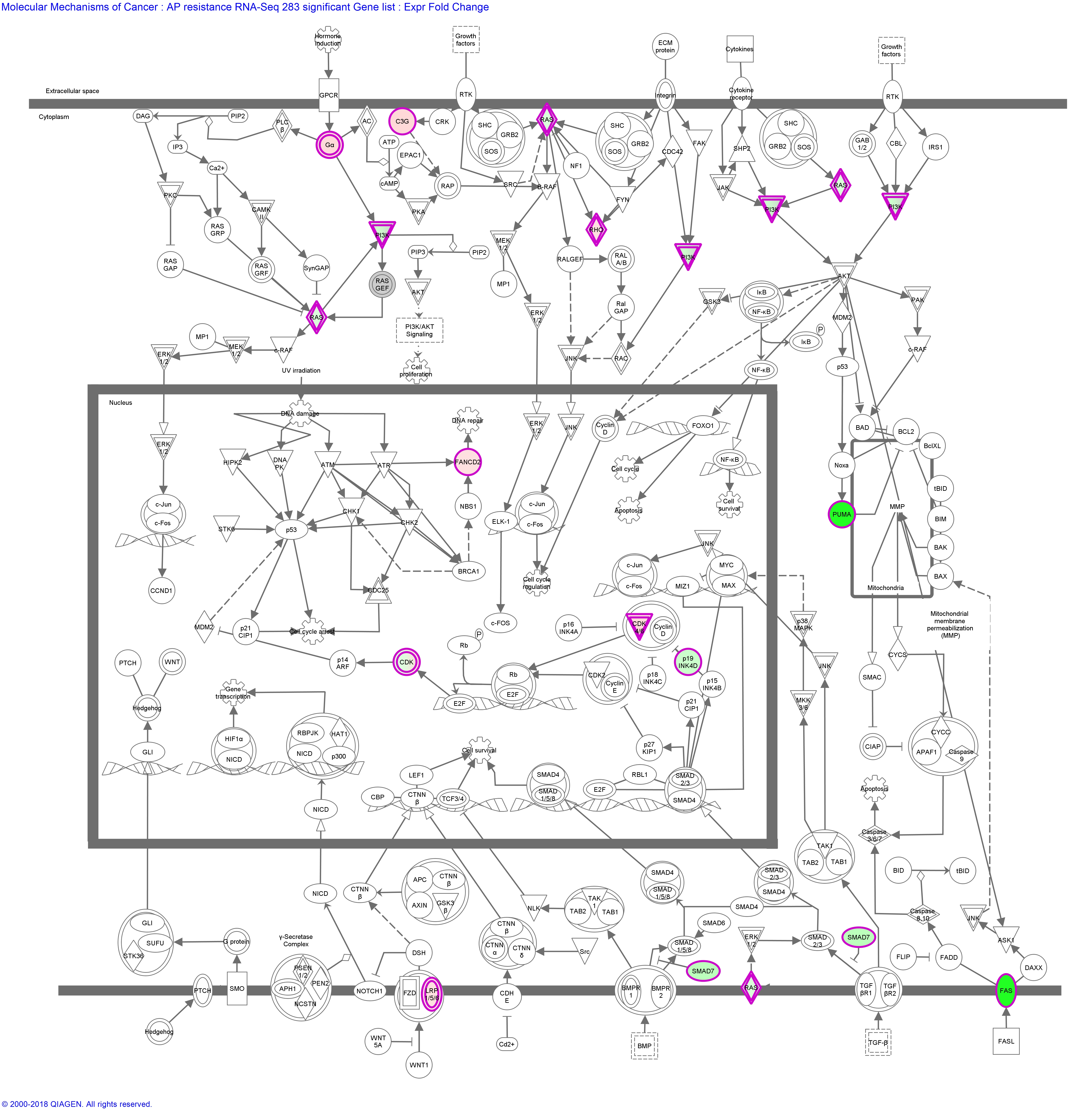
 **Supplemental Figure 1.** Ingenuity Pathways Analysis of relative gene expression data from resistant and parental SCLL cells identifies the cell death pathway as one the most significantly dysregulated with reduction in PUMA levels (Green symbol). Symbols in red indicate upregulation of genes in the resistant cells.
